# Regulation of circulating CTRP-2/CTRP-9 and GDF-8/GDF-15 by intralipids and insulin in healthy control and polycystic ovary syndrome women following chronic exercise training

**DOI:** 10.1186/s12944-021-01463-3

**Published:** 2021-04-19

**Authors:** Jayakumar Jerobin, Manjunath Ramanjaneya, Ilham Bettahi, Raihanath Parammal, Kodappully Sivaraman Siveen, Meis Alkasem, Myint Aye, Thozhukat Sathyapalan, Monica Skarulis, Stephen L. Atkin, Abdul Badi Abou-Samra

**Affiliations:** 1 Qatar Metabolic Institute, Department of Medicine and Academic Health System, Hamad Medical Corporation, Doha, Qatar; 2 Interim Translational Research Institute, Academic Health System, Hamad Medical Corporation, Doha, Qatar; 3 Department of Academic Endocrinology, Diabetes and Metabolism, Hull York Medical School, Hull, UK; 4 RCSI Bahrain, Adliya, Bahrain

**Keywords:** C1Q/TNF related proteins, Growth differentiation factors, Lipid, Insulin, Insulin resistance, Euglycemic clamp, Exercise and polycystic ovary syndrome

## Abstract

**Background:**

Polycystic ovary syndrome (PCOS) is associated with obesity, diabetes, and insulin resistance. The circulating C1Q/TNF-related proteins (CTRP-2, CTRP-9) and growth differentiation factors (GDF-8, GDF-15) contribute to glucose and lipid homeostasis. The effects of intralipids and insulin infusion on CTRP-2, CTRP-9, GDF-8 and GDF-15 in PCOS and control subjects before and after chronic exercise training were examined.

**Methods:**

Ten PCOS and nine healthy subjects were studied at baseline status and after moderate-intensity chronic exercise training (1 h exercise, 3 times per week, 8 weeks). All participants were infused with 1.5 mL/min of saline or intralipids (20%) for 5 h, and during the last 2 h of saline or intralipids infusion hyperinsulinemic-euglycemic clamp (HIEC) was performed. CTRP-2, CTRP-9, GDF-8 and GDF-15 levels were measured at 0, 3 and 5 h.

**Results:**

Intralipids dramatically increased CTRP-2 levels in PCOS (*P* = 0.02) and control (*P* = 0.004) subjects, which was not affected by insulin infusion or by exercise. Intralipids alone had no effects on CTRP-9, GDF-8, or GDF-15. Insulin increased the levels of GDF-15 in control subjects (*P* = 0.05) during the saline study and in PCOS subjects (*P* = 0.04) during the intralipid infusion. Insulin suppressed CTRP9 levels during the intralipid study in both PCOS (*P* = 0.04) and control (*P* = 0.01) subjects. Exercise significantly reduced fasting GDF-8 levels in PCOS (*P* = 0.03) and control (*P* = 0.04) subjects; however, intralipids infusion after chronic exercise training increased GDF-8 levels in both PCOS (*P* = 0.003) and control (*P* = 0.05) subjects and insulin infusion during intralipid infusion reduced the rise of GDF-8 levels.

**Conclusion:**

This study showed that exogenous lipids modulate CTRP-2, which might have a physiological role in lipid metabolism. Since chronic exercise training reduced fasting GDF-8 levels; GDF-8 might have a role in humoral adaptation to exercise. GDF-15 and CTRP-9 levels are responsive to insulin, and thus they may play a role in insulin responses.

## Background

Polycystic Ovary Syndrome (PCOS) is an endocrine and hormonal disorder affecting 15 to 20% of reproductive age women [1, 2]. PCOS causes infertility and is associated with obesity, dyslipidemia, insulin resistance (IR), type 2 diabetes (T2D), and cardiovascular risk [3]. Greater than 50% of the women with PCOS are either obese or overweight, and increased adiposity contributes to decreased sex hormone-binding globulin (SHBG) and elevated androgen production [4, 5]. IR is common among non-obese PCOS; however, obesity aggravates IR and metabolic abnormalities in PCOS women [6–8].

C1Q/TNF-related proteins (CTRPs) are glycoproteins belonging to the adipokine family, predominantly secreted by adipose tissue that regulates lipid and metabolism of glucose [9]. The adipose tissue expression of CTRP-2 is upregulated in ob/ob mice compared to the lean animals [10]. CTRP-2 knockout mice had higher energy expenditure and metabolic rates leading to adipose tissue lipolysis and lower body weight [11]. CTRP-2 transgenic mice have an enhanced ability to handle a lipid challenge and showed improved insulin tolerance relative to littermate animals [12]. In humans, CTRP-9 levels are reported to be elevated in obese individuals and declined following the weight loss surgery [13, 14]. Elevated circulating CTRP6 levels were observed in PCOS subjects, whereas decreased CTRP12 and CTRP13 levels were observed in PCOS compared to non-PCOS subjects irrespective of obesity [15, 16]. The elevated triglyceride-glucose and triglyceride/High-density lipoprotein cholesterol (TG/HDL-c) were associated with IR among PCOS women [17]. In L6 myotubes, recombinant CTRP-9 increases fatty oxidation and reduces lipid accumulation in H4IIE hepatocytes [18].

Growth differentiation factors (GDF), namely GDF-8 and GDF-15, are secreted proteins that belongs to the transforming growth factor-beta (TGF-β) superfamily [19]. GDF8 or myostatin plays a key role in skeletal muscle homeostasis, and GDF-8 deficient mice studies had reduced fat accumulation, increased insulin sensitivity, and glucose uptake [20]. Increased GDF-8 expression was found in follicular fluid and this was linked to a lower pregnancy rate in PCOS women undergoing in vitro fertilization (IVF) [21]. In the third trimester, elevated GDF-15 or macrophage inhibitory cytokine-1 (MIC-1) was observed in Chinese pregnant women [22]. The GDF-15 receptor is known as GDNF family receptor α–like that promotes weight loss in mice [23, 24]. GDF-15 was shown to be elevated in obese IR subjects and correlated with C-peptide, glucose, insulinogenic index and prehepatic beta-cell function suggesting its role in beta cell function [25]. GDF-15 regulates oxidized low-density lipoprotein induced lipid homeostasis and autophagy in human macrophages associated with atherosclerosis development [26].

Both CTRPs and GDFs are key regulators of glucose and lipid homeostasis; this study was designed to understand the interplay between circulatory CTRP-2/CTRP9 and GDF-8/GDF-15 by insulin, lipid and the effect of exercise in healthy women, and subjects with PCOS.

## Methodology

### Ethics statement

All the study participants gave written informed consent, and this study was conducted according to the Good Clinical Practice and Declaration of Helsinki. The study was approved by Medical Research Center, Hamad Medical Corporation (RP #17180/17) and Yorkshire and Humber Ethics Committee, Leeds (10/H1313/44). ISRCTN number: ISRCTN42448814.

### Subject selection

Ten PCOS patients and nine healthy control subjects participated in this study that were BMI and age matched controls. The participants included in the study were non-smokers, not on any regular medications, and did not have any concurrent illness. Control subjects were recruited by adverts within the hospital, whilst PCOS subjects were recruited from the endocrine clinics of Hull Royal Infirmary UK. Rotterdam criteria were used to define PCOS; the presence of two criteria among the three following criteria, hyperandrogenism, oligomenorrhoea and polycystic ovaries on ultrasound [27, 28]. The study participants did not use any drugs with acetylsalicylic acid during the study period. The study participants had a negative pregnancy test before inclusion in the study. The study participants did not modify their dietary habits and lifestyle. Subjects with impaired glucose tolerance based on an OGTT were excluded.

### Hyperinsulinemic euglycemic clamp (HIEC)

PCOS subjects were studied after six or more weeks of amenorrhea, and healthy control were studied during the first menstrual cycle week. The protocol included fasting and infusion of normal saline (1.5 mL/min) for 5 h. Before starting the euglycemic clamp, the blood samples were drawn from the study participants at the 3 h time point (of the 5 h study) for both saline and Intralipid studies. After 3 h of saline infusion, HIEC was started with the infusion of intravenous insulin at a rate of 80 mU/m^2^surfacearea/min for 20 min followed by 40 mU/m^2^surfacearea/min for an additional 100 min. Plasma glucose was clamped at 5 mmol/L using 20%dextrose, the rate of which was adjusted according to the arterialized blood glucose measured every 5 min. A week later, the study participants were infused with intralipid 20% (1.5 mL/min) with 0.3unit/kg/min unfractionated heparin sodium for 5 h. During the last 2 h of intralipid infusion, a 2 h HIEC was performed. Insulin sensitivity (IS) measurement was assessed by calculating the glucose disposal rate (M value, mg/kg/min).

### Chronic exercise training programme

Chronic exercise training was undertaken as explained [29]. Within a week following the baseline assessment, participants began attending 3 supervised exercise sessions per week for 8 weeks. Where possible, each session was 1 h in duration depending on their ability to complete the sessions with no complications. The programme used either a Woodway ELG55 motorized treadmill (Woodway, Weil an rhein, Germany), or a HP Cosmos Pulsar Treadmill (H/P/Cosmos) with the same protocol. Participants performed all sessions on a motorized treadmill working at or as closely as possible to 60% VO_2max._ VO_2_/kg was measured after the warm-up, which lasted for 5 min at 4.5 km^.^h^− 1^ and for 10 min to confirm the appropriate exercise intensity. The intensity of exercise was then adjusted by altering the speed of the treadmill if this value was not within ±2.5% of the target oxygen uptake. Following 10 min gas collection, the facemask was withdrawn with the speed of the treadmill remaining as it was. A further gas collection was made at 40 min to confirm the desired intensity for a 5 min period. If this intensity was out of range, then the treadmill speed was once again altered if required. Heart rate (HR) and rate of perceived exertion (RPE) [30] were monitored every 15 min throughout the session. If the participants felt that they could not continue with the exercise for reasons such as injury or fatigue, they were able to stop at any time if necessary. Likewise, if the participant needed to recover from fatigue, the intensity was reduced for a period of time; otherwise the intensity remained at the level pre-determined. Each session ended with a 5 min cool down at 4.5 km^.^h^− 1^ and participants would then be free to leave once HR returned to within 120% of basal levels. The participants in the study were not asked to alter their diet in any way and were to continue as normal with their calorie consumptions throughout the chronic exercise training programme. During exercise session visits, fluid intake was permitted ad libitum.

**Fig. 1:**
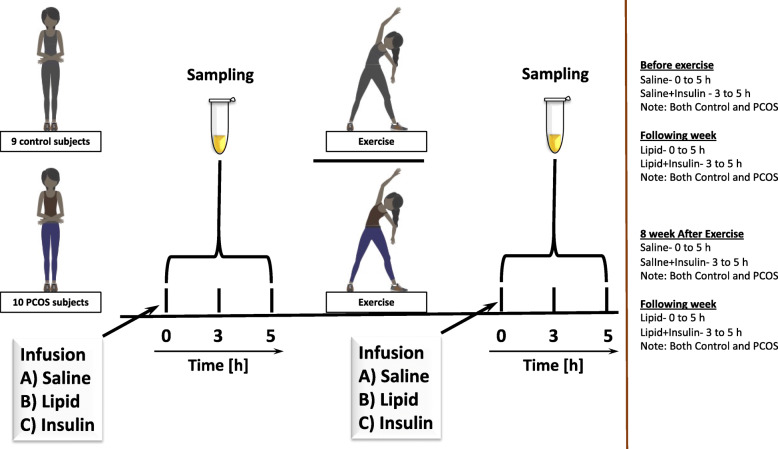
Overall schematic representation of the study. Saline-HIEC and the following week Intralipid-HIEC clamp were performed. All the study participants umderwent eight weeks of chronic exercises training. Following this Saline-HIEC and the following week Intralipid-HIEC clamp were performed to assess the effect of exercise on insulin sensitivity

After 8 weeks chronic exercise training intervention within a week, saline and HIEC clamp were performed, and the following week intralipid and HIEC clamp were performed in all the study participants (Fig. 1).

### Biochemical and hormonal parameters measurement

Plasma glucose, triglycerides (TG), total cholesterol (TCH), HDL, and Alanine transaminase (ALT) were measured by SynchonLX20 analyzer, High Wycombe, UK. LDL-c levels were performed by the Friedwald equation. The enzymatic calorimetric method was used to measure non-esterified fatty acids (NEFA) in Konelab20 autoanalyzer, Swedesboro, NJ, USA. Chemiluminescent immunoassay kit (Euro/DPC, Llanberis, UK) was used to measure insulin levels. The SHBG was measured using DPC immulite 2000 analyzer. Testosterone levels were measured by HPLC/tandem mass spectrometry (Waters Corporation, Manchester, UK). Free androgen index (FAI) was performed by using the formula:100*testosterone/SHBG. Waist to Hip ratio (WHR) is calculated by waist divided by the hip measurement (Waist/Hip).

CTRP-2 was measured using ELISA kits obtained from MyBioSource, San Diego, CA, USA with a minimum detectable dose (MDD) 0.039 ng/mL and detection range 0.156-10 ng/mL (intra-assay CV: < 8% and inter-assay < 10%). CTRP-9 levels were measured by ELISA kits obtained from MyBioSource, San Diego, CA, USA, with sensitivity less than 0.412 ng/mL and detection range 0.78-50 ng/mL (intraassay CV: < 5.9% and inter-assay CV: 9.5%). GDF8/myostatin was measured using a commercially available Quantitine kit obtained from R&D Systems, Minneapolis, MN, USA with minimum detectable dose (MDD) ranged from 0.922–5.32 pg/mL and assay range 31.3-2000 pg/mL (intra-assay CV < 5.4% and inter-assay variation < 6.0). GDF-15 was measured using ELISA kits obtained from Thermo Scientific, Frederick, MD, USA with sensitivity 2 pg/mL and detection range 1.10-800 pg/mL (intra-assay CV < 10% and inter-assay CV < 12%). All the samples measured gave values within the dynamic range of the assays.

### Statistical analysis

Statistical analysis was performed using SPSS 22.0 software and Graphpad Prism 5. Two-way ANOVA with Tukey’s multiple comparison test was performed to indicate significant differences between the variables. The Student’s unpaired t-test was performed to determine the significance between the groups. The Student’s paired t-test was performed to determine the significance within the control and PCOS subjects. The mean difference was calculated from the difference between the values after the chronic exercise training and before chronic exercise training expressed as mean and 95% CI. Pearson correlation coefficient was performed to study the correlations between circulating CTRP2, CTRP9, GDF-8, and GDF-15 with anthropometric and biochemical variables. The data are presented as mean ± standard deviation (SD) for normally distributed variables.

## Results

### Baseline characteristics of CTRP and GDF levels in healthy control and PCOS subjects

**Fig. 2:**
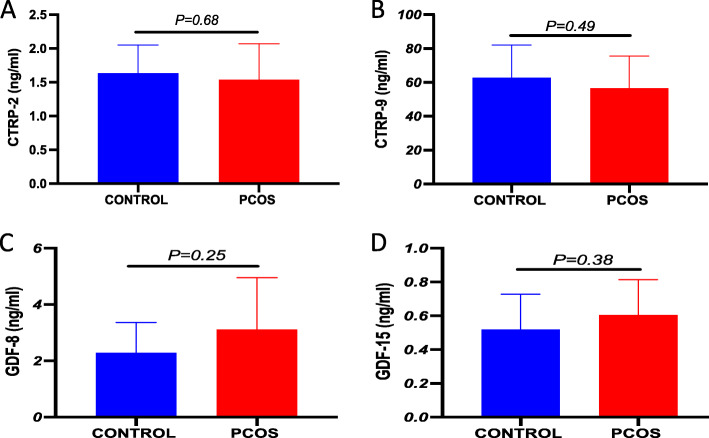
CTRP2, CTRP9,GDF-8 and GDF-15 levels in healthy controls and PCOS women. **a** CTRP-2; **b** CTRP-9; **c** GDF-8; **d** GDF-15. *P* < 0.05 is found to be statistically significant. No significant differences in CTRP-2, CTRP-9, GDF-8 and GDF-15 levels were observed between control and PCOS women. Student’s unpaired t-test was done to determine the significance between the control and PCOS subjects

The baseline characteristics of the study subjects have been previously reported [27, 31]. In brief, the PCOS subjects were overweight, and other clinical variables that were similar between the control and PCOS subjects include BMI, lipid profiles (TCH, TG, HDL, and LDL), SBP, DBP, and FPG. In PCOS subjects, significantly elevated ALT (*P* = 0.03), HOMA-IR (*P* = 0.05), and FAI (*P* = 0.002) whereas decreased SHBG (*P* = 0.001) levels were observed compared to healthy control (Table 1) [27]. Circulating CTRP-2 (*P* = 0.68), CTRP-9 (*P* = 0.49), GDF-8 (*P* = 0.25) and GDF-15 (*P* = 0.38) levels were similar in healthy control and PCOS subjects (Fig. 2).

**Table 1:** Anthropometric, biochemical and hormonal measurement (means + SD) of controls and PCOS subjects

Clinical Variables	Control (***n*** = 9)	PCOS (***n*** = 10)	***P***-value
**Age (years)**	24.8 ± 6.3	28.2 ± 7.1	0.29
**BMI (kg/m**^**2**^**)**	25.4 ± 5.8	29.9 ± 5.4	0.09
**Waist (cm)**	79.6 ± 13.6	91.7 ± 12.7	0.06
**Hip (cm)**	99.1 ± 14.7	109.9 ± 11.8	0.09
**WHR**	0.8 ± 0.1	0.8 ± 0.1	0.24
**SBP (mmHg)**	117.3 ± 6.2	118.8 ± 10.2	0.71
**DBP (mmHg)**	74.2 ± 8.5	76.2 ± 7.8	0.60
**Free androgen index**	1.9 ± 2.1	6.6 ± 3.2	0.002
**SHBG (nmol/L)**	72.6 ± 29.8	26.0 ± 19.5	0.001
**Testosterone (nmol/L)**	0.9 ± 0.3	1.3 ± 0.6	0.12
**Total cholesterol (mmol/L)**	4.6 ± 0.8	4.1 ± 0.7	0.14
**Triglycerides (mmol/L)**	0.8 ± 0.2	1.3 ± 0.8	0.08
**HDL-c (mmol/L)**	1.5 ± 0.5	1.3 ± 0.4	0.29
**LDL-c (mmol/L)**	2.7 ± 0.6	2.3 ± 0.5	0.17
**ALT (IU/L)**	17.3 ± 5.8	29.8 ± 13.9	0.03
**FPG (mmol/L)**	4.7 ± 0.6	4.7 ± 0.5	0.93
**NEFA (μmol/L)**	505.2 ± 180.2	482.9 ± 144.4	0.77
**HOMA-IR**	1.4 ± 0.8	2.7 ± 1.7	0.05

Values are represented as means + SD

*BMI* Body Mass Index, *WHR* Waist Hip ratio, *SBP* Systolic blood pressure, *DBP* Diastolic blood pressure, *SHBG* Sex hormone binding globulin, *TC* Total cholesterol, *LDL-c* Low density lipoprotein cholesterol and *HDL-c* High density lipoprotein cholesterol and *TG* triglyceride, *ALT* alanine transferase, *FPG* fasting plasma glucose, *NEFA* non-esterified free fatty acids, *HOMA-IR* Homeostatic model assessment of insulin resistance

Student’s unpaired t-test was done between control and PCOS subjects

*P* < 0.05 is considered to be statistically significant

### Effects of insulin infusion

The plasma concentration of CTRP-2, CTRP-9, GDF-8, and GDF-15 were stable during the 3 h of saline infusion. Infusion of insulin for 2 h after the 3 h of saline infusion significantly decreased GDF-8 levels in healthy control (*P* = 0.03) and PCOS women (*P* = 0.04) and significantly increased GDF-15 in healthy control (*P* = 0.05) (Table 2). Insulin infusion did not affect either plasma CTRP-2 and CTRP-9 levels in both groups.

**Table 2:** Effects of insulin and intralipid infusion in control and PCOS subjects before exercise

Study Design ➔		Saline Study • Saline: 0 to 5 H • Insulin and glucose: 3 to 5 H		Intralipid Study • Intralipid: 0 to 5 H • Insulin and glucose: 3 to 5 H	
CTRPs & GDFs	Time (H)	Control (***N*** = 9)	PCOS (***N*** = 10)	Control (***N*** = 9)	PCOS (***N*** = 10)
**CTRP2 (ng/mL)**	0	1.6 ± 0.4	1.5 ± 0.5	1.9 ± 0.6	1.7 ± 0.7
	3H	1.6 ± 0.6	1.8 ± 0.7	4.9 ± 2.4^*a,d*^	3.9 ± 2.2^*a,d*^
	5H	1.9 ± 1.0	2.1 ± 1.0	4.7 ± 1.7^*a,d*^	4.4 ± 1.9^*a,d*^
**CTRP9 (ng/mL)**	0	62.8 ± 19.2	56.6 ± 19.1	62.9 ± 23.1	56.9 ± 23.1
	3H	65.3 ± 18.9	58.4 ± 15.4	69.4 ± 23.2	65.4 ± 27.2
	5H	70.3 ± 24.9	57.3 ± 15.7	57.8 ± 22.8^*b*^	53.8 ± 30.9^*b*^
**GDF8 (ng/mL)**	0	2.3 ± 1.1	3.1 ± 1.8	2.8 ± 1.1	2.8 ± 1.2
	3H	2.0 ± 0.8	2.7 ± 1.2	2.8 ± 1.0	2.6 ± 1.1
	5H	1.7 ± 0.7^*a*^	2.3 ± 0.9^*a*^	2.4 ± 0.7^*d*^	2.6 ± 1.0
**GDF15 (ng/mL)**	0	0.5 ± 0.2	0.6 ± 0.2	0.7 ± 0.4	0.7 ± 0.3
	3H	0.8 ± 0.5	0.7 ± 0.2	0.7 ± 0.4	0.7 ± 0.2
	5H	0.9 ± 0.6^*a*^	0.8 ± 0.2	1.2 ± 1.0	1.2 ± 0.8^*b*^

The data are represented as Mean ± SD

Statistical significance (*P* < 0.05) is shown in the table with superscripts *a, b, c* and *d*

*a* compares 3H or 5H versus 0; *b* compares 5H versus 3H; *c* compares PCOS values versus respective control values; and *d* compares intralipid values versus respective saline values

*CTRPs* C1Q/TNF related proteins, *GDFs* Growth differentiation factors

*a, b* and *d:* Student’s paired t-test; *c:* Two-way ANOVA

### Effects of Intralipid and insulin infusion

Intralipid infusion for 3 h significantly increased CTRP-2 levels in healthy control (*P* = 0.004) and PCOS (*P* = 0.02) women; but had no effects on CTRP-9, GDF-8 or GDF15 (Table 2). Co-infusion of insulin at 3–5 h significantly reduced CTRP-9 levels in healthy control (*P* = 0.01) and PCOS (*P* = 0.04) women, increased GDF-15 levels in PCOS women (*P* = 0.04); but had no effects on CTRP-2 levels (Table 2).

### Effect of eight-week chronic exercise training

Chronic exercise training improved the overall fitness of the participants with VO_2_ max improvement [27], but no weight changes were observed following exercise. Chronic exercise training improved insulin sensitivity in both PCOS and control subjects equally, so there was no difference between groups [27, 32].

The effects of chronic exercise training on basal levels of the CTRPs/GDFs were examined. Chronic exercise training significantly reduced basal GDF-8 levels in healthy control (*P* = 0.04) and PCOS subjects (*P* = 0.03) (Table 3) but did not have significant effects on basal CTRP-2, CTRP-9, and GDF-15 levels.

**Table 3:** Effect of exercise on CTRP2, CTRP9, GDF-8 and GDF-15 levels

CTRPs & GDFs	Control Subjects (***N*** = 9)	***P***-value	PCOS Subjects (***N*** = 10)	***P***-value
**CTRP-2, ng/mL**	0.02 (− 0.27, 0.32)	0.85	0.19 (− 0.18, 0.56)	0.28
**CTRP-9, ng/mL**	−2.88 (−31.17, 25.42)	0.82	−2.31 (−14.72, 10.09)	0.68
**GDF-8, ng/mL**	−1.03 (−1.99, − 0.06)	0.04	−1.54 (−2.91, − 0.17)	0.03
**GDF-15, ng/mL**	− 0.01 (− 0.16, 0.14)	0.84	0.03 (− 0.19, 0.25)	0.75

The data are expressed as the difference between the values after and before exercise expressed as mean and (95% CI)

*CTRPs* C1Q/TNF related proteins, *GDFs* Growth differentiation factors

Student’s paired t-test was done to determine the significant difference within control and PCOS subjects respectively

The modulatory effects of chronic exercise training on intralipids and insulin infusions were studied in these subjects (Table 4). The stimulatory effects of intralipid infusion on CTRP-2 observed before chronic exercise training was also seen in the study after chronic exercise training. In addition, intralipid increased the levels of GDF-8 after chronic exercise training in both the healthy control (*P* = 0.05) and PCOS women (*P* = 0.003). The increase in GDF-8 levels observed in PCOS women during infusion of intralipids was significantly decreased due to co-infusion with insulin (*P* = 0.03).

**Table 4:** Effects of insulin and intralipid infusion in control and PCOS subjects after exercise

Study Design ➔		Saline Study • Saline: 0 to 5 H • Insulin and glucose: 3 to 5 H		Intralipid Study • Intralipid: 0 to 5 H • Insulin and glucose: 3 to 5 H	
Clinical Variables	Time (H)	Control (***N*** = 9)	PCOS (***N*** = 10)	Control (***N*** = 9)	PCOS (***N*** = 10)
**CTRP2 (ng/mL)**	0	1.7 ± 0.6	1.7 ± 0.6	1.7 ± 0.5	1.6 ± 0.6
	3H	1.7 ± 0.6	1.8 ± 0.7	3.2 ± 1.4^*a,d*^	3.4 ± 1.2^*a,d*^
	5H	2.1 ± 1.4	2.3 ± 0.8	3.6 ± 1.6^*a*^	3.5 ± 1.1^*a,d*^
**CTRP9 (ng/mL)**	0	59.9 ± 37.4	54.3 ± 23.9	66.1 ± 27.7	65.8 ± 17.5
	3H	80.4 ± 49.8	70.1 ± 17.6	67.7 ± 34.8	67.5 ± 22.3
	5H	74.5 ± 49.2	69.7 ± 24.4	60.1 ± 44.9	59.5 ± 14.9
**GDF8 (ng/mL)**	0	1.3 ± 0.4	1.6 ± 0.6	1.6 ± 0.9	1.6 ± 0.9
	3H	1.2 ± 0.3	1.5 ± 0.4	2.5 ± 0.8^*a,d*^	2.6 ± 1.0^*a,d*^
	5H	1.0 ± 0.3^*a,b*^	1.3 ± 0.5	2.1 ± 0.9^d^	2.2 ± 1.1^*a,b,d*^
**GDF15 (ng/mL)**	0	0.5 ± 0.3	0.6 ± 0.2	0.7 ± 0.2	0.7 ± 0.2
	3H	0.3 ± 0.2^*a*^	0.6 ± 0.5	0.5 ± 0.4	0.5 ± 0.4
	5H	0.5 ± 0.3^*b*^	0.9 ± 0.4^*b,c*^	0.9 ± 0.9	1.1 ± 0.9^*b*^

The data are represented as Mean ± SD

Statistical significance (*P* < 0.05) is shown in the table with superscripts *a, b, c* and *d*

*a* compares 3H or 5H versus 0; *b* compares 5H versus 3H; *c* compares PCOS values versus respective control values at different time point; and *d* compares intralipid values versus respective saline values

*CTRPs* C1Q/TNF related proteins, *GDFs* Growth differentiation factors

*a, b* and *d:* Student’s paired t-test, *c:* Two-way ANOVA

The level of GDF-15 tends to decrease during the 3 h of saline and intralipid infusions after chronic exercise training; the decrease was significant in saline control group (*P* = 0.03); co-infusion of insulin reversed this decline in GDF-15 in the saline control group (*P* = 0.02) and intralipid PCOS women (*P* = 0.01) (Table 4).

### Correlation analysis of CTRP2/ CTRP9 and GDF-8/GDF-15 with covariates

The CTRP-2/9 and GDF-8/15 association with baseline biochemical, clinical, and hormonal parameters was performed using the Pearson correlation coefficient (Table 5). In PCOS women, CTRP-2 levels correlated positively with testosterone (*r* = 0.71, *P* = 0.02) and CTRP-9 correlated negatively with GDF-15 (*r* = − 0.77, *P* = 0.01). In healthy controls, CTRP-2 negatively correlated with WHR (*r* = − 0.75, *P* = 0.02).

**Table 5:** Pearson correlation coefficient of CTRP2, CTRP9, GDF-8 and GDF-15 with anthropometric and biochemical variables

CLINICAL VARIABLES	CTRP-2		CTRP-9		GDF-8		GDF-15	
	Control (***N*** = 9)	PCOS (***N*** = 10)	Control (***N*** = 9)	PCOS (***N*** = 10)	Control (***N*** = 9)	PCOS (***N*** = 10)	Control (***N*** = 9)	PCOS (***N*** = 10)
	*r*	*r*	*r*	*r*	*r*	*r*	*r*	*r*
**CTRP-2**	1	1	−0.49	−0.01	−0.02	0.16	−0.43	0.07
**CTRP-9**	−0.49	−0.01	1	1	−0.001	0.58	0.44	− 0.77*
**GDF-8**	−0.02	0.16	−0.001	0.58	1	1	−0.17	−0.46
**GDF-15**	−0.43	0.07	0.44	−0.77*	−0.17	− 0.46	1	1
**Age**	−0.60	−0.33	0.41	−0.03	0.30	−0.24	0.59	−0.40
**BMI**	−0.29	0.39	−0.35	−0.09	− 0.10	0.20	0.55	0.08
**Waist**	−0.24	0.38	−0.38	−0.003	− 0.01	0.14	0.51	0.10
**Hip**	0.05	0.48	−0.62	−0.15	− 0.14	0.02	0.35	0.19
**WHR**	−0.75*	0.05	0.45	0.21	0.26	0.24	0.46	−0.08
**SBP**	−0.26	0.19	−0.18	−0.58	0.14	−0.50	0.48	0.65*
**DBP**	0.21	0.06	−0.50	−0.39	0.10	0.24	0.38	0.51
**Testosterone**	−0.09	0.71*	0.16	0.24	−0.12	0.64*	−0.40	−0.12
**FAI**	−0.04	0.31	−0.34	0.06	−0.52	0.39	−0.11	−0.44
**SHBG**	0.14	−0.05	0.45	−0.20	0.30	−0.22	− 0.15	0.62
**TC**	0.05	−0.01	0.22	0.40	0.26	0.46	0.44	−0.34
**TG**	−0.39	−0.13	0.16	0.35	0.41	−0.08	0.57	−0.15
**HDL**	0.20	−0.25	0.23	0.23	0.52	−0.12	−0.30	− 0.29
**LDL-c**	−0.24	0.24	0.11	0.17	−0.06	0.74*	0.81*	−0.15
**FPG**	−0.20	−0.37	− 0.15	−0.51	− 0.12	−0.26	0.46	0.68*
**ALT**	0.03	0.37	−0.35	0.04	0.45	0.41	−0.26	0.04
**NEFA**	0.16	−0.56	− 0.33	−0.47	0.47	−0.27	0.10	0.34
**HOMA-IR**	−0.62	0.002	−0.23	0.12	−0.42	0.56	0.27	0.005

*r* is Pearson correlation coefficient

*CTRPs* C1Q/TNF related proteins, *GDFs* Growth differentiation factors, *BMI* Body Mass Index, *WHR* Waist Hip ratio, *SBP* Systolic blood pressure, *DBP* Diastolic blood pressure, *SHBG* Sex hormone binding globulin, *TC* Total cholesterol, *LDL-c* Low density lipoprotein cholesterol and *HDL-c* High density lipoprotein cholesterol and *TG* triglyceride, *ALT* alanine transferase, *FPG* fasting plasma glucose, *NEFA* non-esterified free fatty acids, *HOMA-IR* Homeostatic model assessment of insulin resistance

* *P < 0.0*5 is considered to be statistically significant

GDF-8 correlated positively with testosterone (*r* = 0.64, *P* = 0.05) levels and LDL-c (*r* = 0.74, *P* = 0.01) in PCOS women. However, GDF-15 correlated positively with LDL-c (*r* = 0.81, *P* = 0.01) in healthy controls. In PCOS women, GDF-15 correlated positively with SBP (*r* = 0.65, *P* = 0.04) and FPG (*r* = 0.68, *P* = 0.03).

## Discussion

Infusion of lipids during HIEC is reported to shift the fuel source from glucose to lipids for cellular oxidative processes. The dramatic increase in TG reverses insulin inhibition of lipid oxidation and blunts insulin-stimulated glucose utilization [33, 34] that is consistent with the finding of reduced IS during intralipid infusion. Elevated FFA levels following intralipid administration are secondary to lipase-mediated hydrolysis of TG. Since lipase is inhibited by insulin, FFA levels were reduced during the HIEC in all subjects both during saline and intralipid infusion [35, 36] that is in accord with previous reports that insulin reduced NEFA in both groups during the first study period and during the intralipid infusion [31].

Intralipid infusion increases the plasma levels of CTRP-2 and raises the possibility that CTRP-2 may be regulated by endogenous lipid, or that CTRP-2 may play a physiological role in lipid metabolism. Consistent with these hypotheses is the finding in animal models of CTRP-2 deficiency [11] and CTRP-2 overexpression [12]. CTRP-2 knock-out mice showed elevated energy expenditure, and upregulation of lipolytic enzymes [11] whereas CTRP-2 transgenic mice subjected to diet-induced obesity showed improved lipid and insulin tolerance [12]. In response to lipid infusion, CTRP-2 transgenic mice have a greater capacity to clear an acute increase in FFA compared to WT controls. During the fasting state, CTRP-2 transgenic mice can mobilize FFA in adipose tissue via the b3-adrenergic receptor [12]. This suggests that in humans, CTRP-2 might play a physiological role in lipid and energy metabolism: and that a negative feedback mechanism between TG and CTRP-2 may physiologically regulate their concentrations.

In cultured myotubes, CTRP-2 activates AMPK and acetyl-coA resulting in glycogen accumulation, glucose uptake, and fatty acid oxidation [12, 37]. An insulin tolerance test (ITT) in CTRP-2 transgenic mice shows an enhanced glucose disposal rate indicating improved insulin action compared to wild-type (WT) mice [12]. The insulin-stimulated glucose disposal rate was lower in PCOS subjects during lipid infusion and an acute increase in NEFA with intralipids lowered the glucose disposal rate in the skeletal muscle indicating IR in PCOS subjects compared to BMI and age-matched controls [27]. Lifestyle modifications with increased physical activity reduce metabolic complications in PCOS subjects [38]. During the lipid infusion, women with PCOS who had undertaken the exercise training had an improved glucose disposal rate. The improvement in IR following chronic exercise training is reduced fat metabolites and increased fat oxidation [27, 32]. No changes in CTRP-2 levels were seen following chronic exercise training, but this was not surprising since no body weight changes were observed, and CTRP-2 levels are related to changes in body weight [12]. As CTRP-2 is not affected by insulin levels then the change in insulin sensitivity would also not affect CTRP-2 levels.

Circulating CTRP-9 levels are significantly increased in patients with T2D and strongly associated with IR [13]. CTRP-9 levels were correlated positively with serum concentrations of LDL-c and total cholesterol in PCOS subjects [39]. Elevated CTRP2 and decreased CTRP9 are associated with the risk and progression coronary artery disease [40–42]. In this study we did not observe any significant changes of CTRP-2 and CTRP-9 in either controls or PCOS subjects. Targeted CTRP-9 deletion in animal models resulted in increased fasting insulin levels, decreased insulin sensitivity, and food intake. Overexpression of CTRP-9 resulted in improved insulin sensitivity by reducing in fasting glucose and insulin levels [18]. CTRP-9 deletion resulted in decreased AMPK activation in skeletal muscles [43]. Treatment with recombinant CTRP-9 enhanced fatty oxidation through the reduced accumulation of lipid accumulation and AMPK activation in hepatocytes [18]. This study on human did not show any differences in CTRP-9 levels between control and PCOS women or significant changes in CTRP-9 levels after insulin or intralipid infusion; Therefore, this study did not support an important role for CTRP-9 in human subjects in terms of homeostasis during HIEC, hypertriglyceridemia, and chronic exercise training.

In PCOS women, increased GDF-8 levels were associated with increased BMI, FPG and decreased LDL-c was associated with increased GDF-8 levels [44, 45]. The increased skeletal muscle and circulating GDF-8 levels are due to physical inactivity, resulting in metabolic deterioration leading to the progression from IR to T2DM [46, 47]. Aerobic exercise decreases the skeletal and circulatory GDF-8 levels, thus improving IR in obese and T2DM subjects [47–49]. Similarly, decreased GDF-8 levels after chronic exercise training were observed in both healthy control and PCOS women. GDF-8 treatment suppresses adipocyte differentiation by inhibiting lipid accumulation and promoting the secretion of lipolytic enzymes [50]. GDF-8 inhibition in skeletal muscle improves dyslipidemia and insulin sensitivity thereby preventing the development of diabetes in lipodystrophy mouse model [51]. The reduced GDF-8 levels following chronic exercise training therapy were elevated by the intralipid infusion and insulin infusion reduced GDF-8 levels in PCOS women suggesting a role of GDF-8 in insulin sensitivity.

GDF-15 significantly correlated with age and HOMA-IR in PCOS women [52]. In this study, GDF-15 was associated positively with LDL-c in healthy control and FPG in PCOS women. In T2DM and obese subjects, chronic exercise training therapy increases circulatory GDF-15. It is associated with increased β-cell disposition, insulin sensitivity, and fat mass reduction [53, 54], but no significant changes in GDF-15 were observed after chronic exercise training. Fasting and a ketogenic diet exacerbates hepatic GDF-15 levels promoting fatty acid oxidation and ketogenesis in the liver mouse model [55]. Fasting is associated with decreased GDF-15 levels, but oral glucose ingestion increased GDF-15 levels back to baseline in both lean and obese human subjects. In HepG2 cells, insulin and glucose significantly stimulated GDF-15 secretion and transcription [56]. Hyperinsulinemia increases circulatory GDF-15 during different stages of adiposity [57]. Co-infusion of intralipids with insulin significantly increased GDF-15 levels in PCOS subjects.

### Study strength and limitations

The major strength was that this is the first study performed in humans to study the relationship between insulin and lipid infusion on circulating CTRP-2, CTRP-9, GDF-8, and GDF-15. This study was an interventional study with a standard methodology for characterizing the PCOS patients with well-supervised exercise that was a significant strength. The smaller study group is one of the limitations of this study; therefore, the results are to be cautiously interpreted. In large cohorts, these sorts of interventional studies will be challenging to perform because of frequent blood sampling during the clamp and multiple visits. Only Caucasians were included as participants, which could have limited the findings of this study.

## Conclusion

This is the first study performed in humans to show that intralipid infusion is a potent stimulator for CTRP-2 in healthy control and PCOS subjects, suggesting that CTRP-2 might have a physiological role in lipid metabolism. The study also shows that 8 weeks of chronic exercise training decreases GDF-8 in healthy control and PCOS women suggesting that GDF-8 regulation may have a role in adaptation to exercise, and GDF-15 and CTRP-9 may play a role in the insulin response. Based on the findings, it appears that future studies could focus on understanding the relationship between CTRP2 in dyslipidemia. The complex interactions between insulin and intralipids on CTRPs/GDFs in human suggest that these factors play an important role in the overall metabolic responses to metabolic challenges.

## Data Availability

The data are available upon request from the corresponding author.

## Abbreviations

ALT: Alanine transaminase
BMI: Body mass index
CTRP: C1Q TNF related protein
DBP: Diastolic blood pressure
FAI: Free Androgen Index
FFA: Free fatty acids
FPG: Fasting plasma glucose
GDF: Growth differentiation factors
HDL: High density lipoprotein
HIEC: hyperinsulinemic-euglycemic clamp
HOMA-IR: Homeostatic model assessment of insulin resistance
LDL: Low density lipoprotein
IR: Insulin resistance
NEFA: Non-esterified fatty acids
OGTT: Oral glucose tolerance test
PCOS: Polycystic ovary syndrome
SBP: Systolic blood pressure
SHBG: Sex hormone binding globulin
T2DM: Type-2 diabetes mellitus
TCH: Total cholesterol
TG: Triglycerides
ITT: Insulin tolerance test
WT: wild-type
